# Optical Non-Invasive Glucose Monitoring Using Aqueous Humor: A Review

**DOI:** 10.3390/s25134236

**Published:** 2025-07-07

**Authors:** Haolan Xi, Yiqing Gong

**Affiliations:** 1 Department of Ophthalmology, Affiliated People’s Hospital, Jiangsu University, Zhenjiang 212002, China; xihaolan@163.com; 2 Zhenjiang Kangfu Eye Hospital, Zhenjiang 212002, China; 3 School of Medicine, Xiamen Eye Center and Eye Institute of Xiamen University, Xiamen 361102, China

**Keywords:** non-invasive, glucose biosensor, optics, Raman spectroscopy, polarization, infrared, spectroscopy, spectrometer

## Abstract

This review explores optical technologies for non-invasive glucose monitoring (NIGM) using aqueous humor (AH) as media, addressing the limitations of traditional invasive methods in diabetes management. It analyzes key techniques such as Raman spectroscopy, polarimetry, and mid- and near-infrared spectral methods, highlighting their respective challenges, alongside emerging hybrid approaches like photoacoustic spectroscopy and optical coherence tomography. Crucially, the practical realization of these optical methods for portable NIGM hinges on advanced instrumentation. Therefore, this review also details progress in compact NIR spectrometers. While conventional systems often lack suitability, significant advancements in on-chip technologies—including miniaturized dispersive spectrometers and various on-chip Fourier transform systems (e.g., spatial heterodyne, stationary wave integral, and temporally modulated FT systems)—utilizing integration platforms like SOI and SiN are promising. Such innovations offer the potential for high spectral resolution, large bandwidth, and miniaturization, which are essential for developing practical AH-based NIGM systems to improve diabetes care.

## 1. Introduction

### 1.1. Diabetes Mellitus: Pathogenesis and Categorization

Diabetes mellitus (DM) is a metabolic disorder characterized by high blood glucose (hyperglycemia) due to defects in insulin production or action. This condition is a major global health issue, leading to severe long-term complications affecting the cardiovascular, renal, neurological, and visual systems, as cited in [1,2,3,4]. The two primary forms are Type 1 DM [5], an autoimmune condition causing a total lack of insulin, and Type 2 DM [6,7], which involves insulin resistance and is often linked to lifestyle factors. According to the latest data from the International Diabetes Federation’s Diabetes Atlas (11th Edition), an estimated 589 million adults are currently living with diabetes globally as of 2025. This represents a substantial rise from the 537 million reported in the 10th edition, which used 2021 data. Projections from the IDF paint an even grimmer picture, forecasting that the number of adults with diabetes will soar to 853 million by 2050 [8].

Sustained hyperglycemia damages blood vessels, significantly increasing the risk of heart attacks and strokes [9], kidney disease (nephropathy) [10], nerve damage (neuropathy) [11], and eye conditions [12,13].

Current “gold-standard” glucose monitoring relies on invasive blood sampling, such as fingerstick tests and venous plasma assays [14]. These methods are often painful, inconvenient, and prone to inaccuracies. Crucially, their intermittent nature fails to capture important glycemic fluctuations, like post-meal spikes and nocturnal hypoglycemia, which are linked to complications. An individual’s glucose sensitivity determines how effectively their body manages glucose after a meal, and low sensitivity significantly increases the long-term risk of developing Type 2 diabetes [6,7].

The impact of diabetes on ocular health is particularly notable, increasing the risk of sight-threatening conditions such as diabetic retinopathy, cataracts, and glaucoma [15,16]. All three conditions are linked to the damaging effects of high and unstable glucose levels. Diabetic retinopathy involves damage to the retina’s fragile blood vessels, leading to leakage and vision loss. For those with cataracts, excess glucose accelerates the clouding of the eye’s lens. Also, diabetes doubles the risk of glaucoma, a disease that damages the optic nerve, often due to increased eye pressure.

The cornerstone of managing diabetes and preventing its complications is maintaining tight glucose control. Non-invasive glucose monitoring (NIGM) is emerging as a transformative technology to achieve this, offering a proactive approach to improve overall well-being [17,18,19]. By enabling the continuous, pain-free tracking of glucose levels, NIGM systems can provide a complete picture of an individual’s glycemic trends, empowering them to protect against all major diabetic complications, including ocular, nerve, kidney, and cardiovascular diseases. To make this a clinical reality, current research is focused on various promising approaches. Among these, optical techniques targeting the eye’s AH are particularly noteworthy, given the close correlation between its glucose levels and those in the blood. This review will explore the advancements and challenges in developing these NIGM technologies for widespread clinical use.

### 1.2. The Overview of Non-Invasive Glucose Detection Technologies

The pursuit of NIGM has spurred significant innovation in sensing technologies designed to replace traditional blood-based tests. We divide NIGM methods into the following two main categories.

#### 1.2.1. Spectroscopic and Optical Techniques

Several techniques rely on the principles of spectroscopy and light interaction:

- Raman spectroscopy identifies the distinct molecular vibrations of glucose through inelastic light scattering.
- Polarimetry takes advantage of glucose’s chiral nature, meaning it rotates the plane of polarized light. The angle of this rotation is directly proportional to the glucose concentration in the light’s path.
- Near-infrared (NIR) and mid-infrared (MIR) spectroscopy quantify levels of glucose by measuring its characteristic absorption of light at specific wavelengths.
- Photoacoustic spectroscopy (PAS) is based on the photoacoustic effect, where absorbed light energy is converted into sound waves, which can be analyzed to determine glucose concentrations.
- Optical coherence tomography (OCT) detects subtle changes in the optical properties of biological tissues that are influenced by varying glucose levels.
- Thermal emission spectroscopy analyzes the body’s natural infrared radiation. The characteristics of this emitted energy are affected by the concentration of substances like glucose.

#### 1.2.2. Wearable and Implantable Sensors

Another major area of NIGM research involves sensors that are worn on or implanted in the body:

- Fluorescence-based sensors typically use small, implantable devices containing molecules that fluoresce, or glow, in the presence of glucose.
- Smart contact lenses are being developed with tiny, flexible sensors that continuously measure glucose concentrations in tears via an enzyme-based reaction.
- Wearable patches adhere to the skin and analyze glucose in sweat. These often use reverse iontophoresis to draw interstitial fluid to the surface for measurement by an electrochemical sensor.
- Saliva-based sensors represent an emerging field, with research focused on creating devices, such as mouthguards, that can detect the low levels of glucose present in saliva.

In this paper, we are focusing on using AH as the sensing medium; hence, we mainly focus on spectroscopic and optical techniques: Raman spectroscopy; polarimetry; near-infrared (NIR); and mid-infrared (MIR). We also discuss the limitations of PAS and OCT when dealing with AH.

## 2. Aqueous Humor: A Biosensing Window for Glucose Monitoring

**Figure 1:**
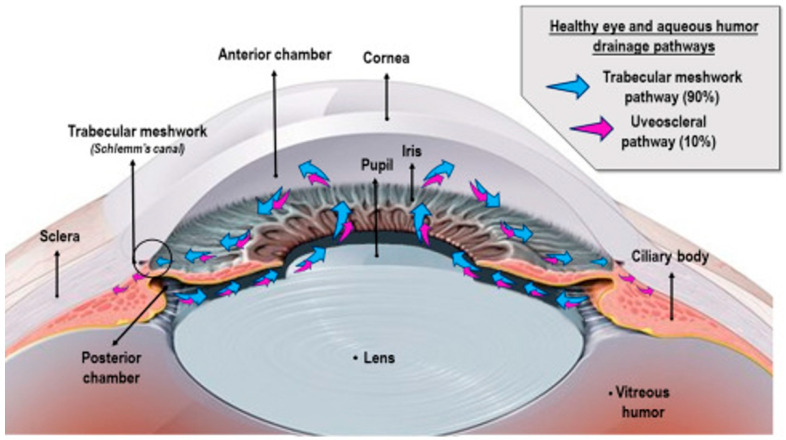
Aqueous humor drainage in healthy eyes through the trabecular meshwork pathway (blue arrows) and uveoscleral pathway (pink arrows) is demonstrated. Adapted with permission [28]. Copyright 2023, ScienceDirect.

The suitability of AH for NIGM is attributed to its distinctive biosynthesis and biochemical makeup. AH synthesis occurs within the non-pigmented epithelium (NPE) of the ciliary body through a continuous secretory process, which is regulated by three interconnected mechanisms [20], as shown in Figure 1. These include (1) passive diffusion driven by electrochemical gradients, (2) ultrafiltration through fenestrated capillaries, and (3) active transport facilitated by Na^+^/K^+^-ATPase and various carbonic anhydrase isoforms [21]. The latter process accounts for 85–90% of AH production [22,23], consuming approximately 3.2 mmol ATP/L to maintain an osmotic gradient of 3–5 mOsm/kg between plasma and AH [24,25,26,27].

Biochemically speaking, AH consists of 98.2% water, with electrolyte concentrations that mirror those in plasma (Na^+^ 142 mM, K^+^ 4.2 mM, Cl^−^ 112 mM), but demonstrates striking hypoproteinemia (total protein < 0.2 mg/mL vs. plasma 70 mg/mL) [29]. This optical clarity is preserved through selective molecular sieving at the blood–aqueous barrier (BAB)—a tripartite structure consisting of NPE tight junctions (claudin-3/ZO-1 complexes), iris vascular endothelium, and Schlemm’s canal trabeculae. The BAB’s molecular weight cutoff (~65 kDa) effectively excludes albumin and immunoglobulins while permitting transcellular glucose transport via GLUT1/GLUT3 facilitative transporters [30,31,32,33].

Glucose equilibration across the BAB follows Michaelis–Menten kinetics, with human ciliary epithelium demonstrating a Vmax of 12.8 nmol·min^−1^·mg^−1^ protein and a Km of 5.2 mM for D-glucose. This transport system maintains a consistent AH/blood glucose ratio (0.68 ± 0.11 in normoglycemia), though pathological hyperglycemia (>11 mmol/L) increases BAB permeability through VEGF-mediated tight junction remodeling [32,33,34]. Diabetic subjects exhibit elevated AH glucose ratios (0.79 ± 0.15 vs. controls 0.63 ± 0.09, *p* < 0.01), correlating with HbA1c levels (r = 0.82) [34].

The AH–blood glucose temporal relationship follows first-order kinetics, characterized by a delay constant (τ) of less than 5 min in primates [30,35]. This equilibration lag arises from two countercurrent processes: anterior chamber turnover (residence time = 100 ± 20 min) and glucose diffusion across the 8–10 μm thick NPE layer (apparent permeability coefficient Papp = 4.1 × 10^−6^ cm/s). Comparative analyses reveal AH glucose tracks systemic levels faster than interstitial fluid (ISF) [30,35,36].

The ocular microenvironment offers inherent advantages for optical biosensing. In contrast, cutaneous tissues suffer from heterogeneous scattering, with a reduced scattering coefficient μ_s_ ≈ 1.2–2.5 mm^−1^ @ 800 nm [37,38,39], and the cornea and AH form a low-turbidity optical pathway (μ_s_ < 0.1 mm^−1^) [40], enabling >90% transmission of visible-to-near-infrared wavelengths (400–1300 nm). This transparency permits the non-contact interrogation of AH glucose using optical methodology. Furthermore, AH’s chemical stability—maintained by active ion transport (Na^+^/K^+^-ATPase) and minimal protein content (<0.1 mg/mL)—reduce nonspecific signal interference compared to blood or tear film [21,30].

## 3. Optical Modalities for Non-Invasive Glucose Monitoring in Aqueous Humor: A Comprehensive Review

The distinctive optical characteristics and ease of access of AH have prompted extensive research into utilizing various optical methods for NIGM. This section offers a thorough overview of the current progress in these endeavors, with a particular emphasis on Raman spectroscopy, mid-infrared and near-infrared absorption spectral data, polarimetry, PAS, and OCT.

### 3.1. Raman Spectroscopy for Aqueous Humor Glucose Detection

#### 3.1.1. Principles of Raman Spectroscopy and Its Application to Glucose Detection

**Figure 2:**
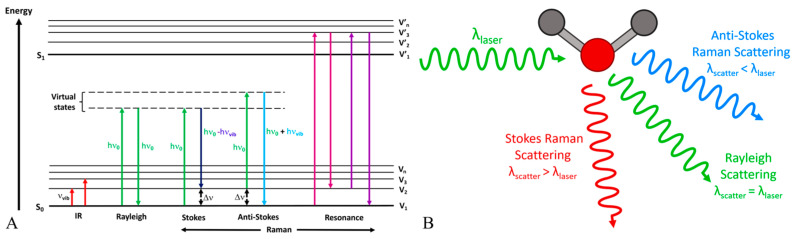
(**A**) Jablonski energy diagram illustrating the transitions involved during infrared absorption, Rayleigh, Raman Stokes, anti-Stokes, and resonance Raman scattering as reported by Geraldes. Adapted with permission [44]. Copyright 2020, MDPI. (**B**) Molecular interaction with light. Adapted from Ref. [45].

**Figure 3:**
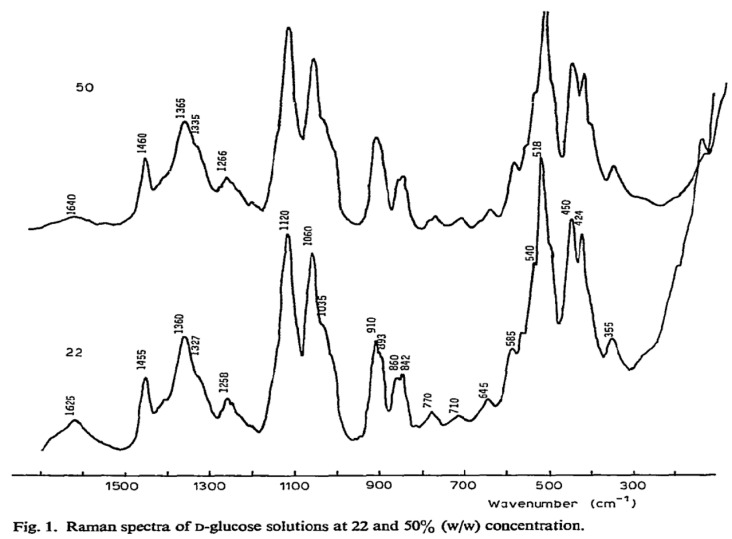
Raman spectra of D-glucose solutions at 22 and 50% (w/w) concentration. Adapted with permission [46]. Copyright 1980, ScienceDirect.

Raman spectroscopy is a spectroscopic method that utilizes the inelastic scattering of light, referred to as the Raman effect, to offer insights into vibrational, rotational, and other low-frequency modes within a system [41,42]. When a monochromatic light source, such as a laser, meets a molecule, the majority of photons undergo elastic scattering (Rayleigh scattering), retaining the same energy (frequency/wavelength) as the incoming photons. A small proportion of the incident photons undergo scattering at frequencies that are typically lower than the incident photons, as illustrated in Figure 2A. The Raman shift represents the difference in frequency, which corresponds to the energy disparity between vibrational levels of the molecule, as depicted in Figure 2B. Raman spectroscopy offers a distinct fingerprint of a sample’s chemical composition, including glucose, due to each molecule possessing a unique set of vibrational modes. Raman spectroscopy can detect glucose by identifying characteristic spectral peaks corresponding to specific vibrational modes of the glucose molecule, as shown in Figure 3. This technique enables quantitative analysis of the glucose concentration in a sample without requiring labeling or reagents. Advanced techniques in data processing, such as multivariate statistical methods and machine-learning algorithms, are frequently utilized to improve the sensitivity and accuracy of glucose measurements derived from Raman spectra, particularly in complex biological matrices [43].

#### 3.1.2. Advantages of Aqueous Humor for Raman Spectroscopy Detection Versus Other Human Tissue

The AH offers numerous advantages as a substrate for glucose detection via Raman spectroscopy, in comparison to other biological tissues such as skin or blood. Firstly, the glucose concentration in AH closely correlates with blood sugar levels, as outlined in the above section, with only a minor delay, thereby serving as a significant indicator of systemic glucose levels. Secondly, the primary constituent of AH is water, exhibiting relatively high optical transparency within the near-infrared spectrum, which is frequently utilized in biological Raman spectroscopy to minimize autofluorescence and tissue injury. This transparency facilitates the efficient excitation of glucose molecules, and the collection of Raman scattered light, with minimal interference from the surrounding medium. Thirdly, AH has a relatively straightforward biochemical composition in comparison to blood or interstitial fluid, with fewer additional organic substances present that could demonstrate Raman scattering and potentially disrupt the detection and quantification of glucose. The Raman characteristic peaks of lactate, ascorbate, and urea in AH are generally distinguishable from those of glucose. This distinction facilitates the accurate calibration and measurement of glucose concentration without significant spectral interference. Additionally, the anterior chamber of the eye allows for non-invasive optical examination through the cornea, thereby bolstering its appropriateness for Raman spectroscopy-based glucose monitoring.

However, the challenge of background fluorescence in vivo Raman spectroscopy can be quite annoying background noise, and it can be effectively managed using various strategies [47]. Instrumental approaches like Shifted-Excitation Raman Difference Spectroscopy (SERDS) [48,49] and time-gated Raman spectroscopy physically separate the Raman signal from fluorescence [50,51]. Also, using near-infrared (NIR) excitation can further minimize its generation. Post-acquisition, computational methods such as polynomial fitting, derivative spectroscopy, and other advanced algorithms are employed to mathematically subtract the residual background. These combined techniques significantly improve the quality and reliability of in vivo Raman data, overcoming a primary limitation of the technology.

#### 3.1.3. Review of the Literature on and Raman Spectroscopy for Aqueous Humor Glucose Detection

Raman spectroscopy’s potential for non-invasive glucose monitoring in AH has garnered significant research attention.

In 1994, initial research established the viability of utilizing Raman spectroscopy to identify and distinguish glucose and lactate in AH [52]. The application of Raman spectroscopy to this particular biological fluid represented a novel innovation, underscoring its promise for metabolic analysis. The study was constrained by contemporary technology, featuring prolonged acquisition times and the requirement for high laser power, rendering it unsuitable for in vivo applications.

Pelletier et al.’s innovation was the use of artificial aqueous humor (AAH) solutions for partial least squares (PLS) model calibration, aiming to create a more robust and reproducible method [53]. The study utilized a high laser energy level (approximately 15 J) for calibration spectra to achieve good signal-to-noise ratios. The researchers also examined the impact of reducing laser energy for collecting human AH spectra on glucose prediction. While acknowledging that challenges remained before this method could be applied in vivo, the study’s findings supported the feasibility of using a calibration model based on high signal-to-noise spectra to successfully predict appropriately gathered low-laser-power spectra for Raman spectroscopic determination of glucose in human eyes.

**Figure 4:**
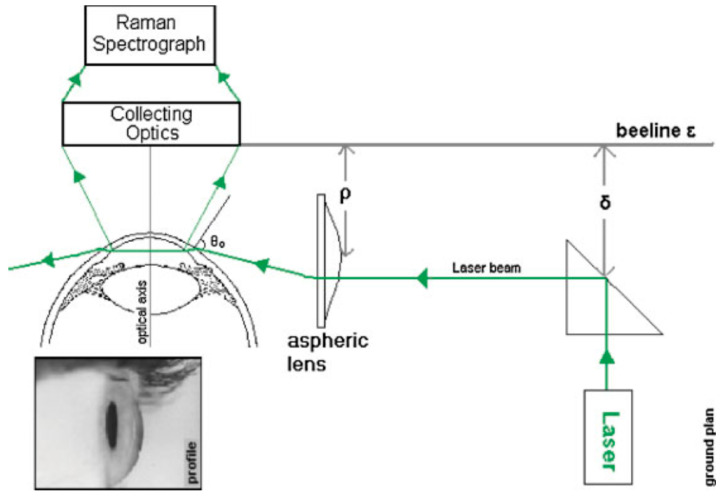
Laser light delivery probe for favorable collection of the Raman photons at 90° scattering geometry scanning the anterior chamber of the eye (ground plane vision). The laser beam inside the anterior chamber is delivered perpendicularly to the optical axis of the eye for safety reasons and Raman signal optimization. Adapted with permission [54]. Copyright 2006, John Wiley and Sons.

In 2006, a significant innovation emerged: the development of a novel laser light delivery probe [54]. This probe was specifically designed to enhance Raman signal collection while minimizing potential harm to ocular tissues. This probe provided a safer and more efficient method for conducting Raman spectroscopy on the eye, as illustrated in Figure 4. The research showed the probe’s capability for detecting drugs and glucose in vitro. However, additional research was required to confirm its safety and effectiveness for human in vivo applications.

**Figure 5:**
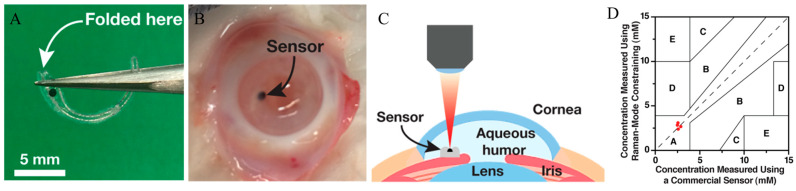
(**A**) Photo of the folded SERS-disk-mounted implant. (**B**) Photo of the implant inserted inside the anterior chamber of an ex vivo rabbit eye. (**C**) Schematic illustration of the glucose measurement made using the SERS implant inside the anterior chamber of an ex vivo rabbit eye. (**D**) Clarke-error-grid analysis of the glucose measurements using our Raman-mode-constraining approach in ex vivo rabbit eyes. All the data measured by the implanted sensor were located in region A of the Clarke error grid, which is labeled as red dots. Adapted with permission [55]. Copyright 2018, American Chemical Society.

Yang et al. presented a novel method utilizing SERS and Raman-mode constraint to improve sensitivity and selectivity for glucose detection [55]. The proposed method monitored changes in Raman peaks induced by glucose binding, providing a more dependable metric than intensity-based SERS. The creation of a miniaturized SERS implant, depicted in Figure 5A, represented a notable advancement. The ex vivo findings were encouraging, yet the technology remained in the developmental phase. Challenges persisted in adapting this approach into a clinically feasible, implantable device capable of long-term continuous glucose monitoring in humans.

**Figure 6:**
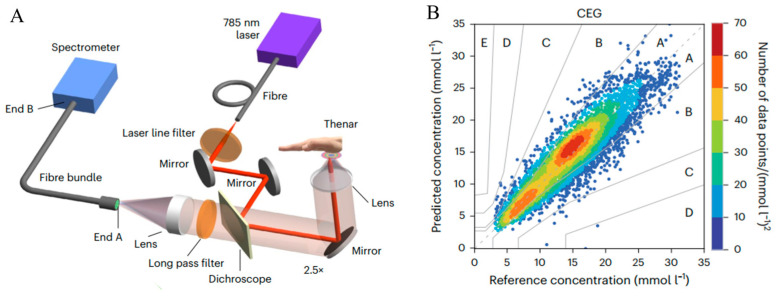
(**A**) Schematic of mμSORS system. mμSORS setup (top). (**B**) CEG of predictions from the PLS regression model in the cross-validation. Adapted with permission [43]. Copyright 2025, Springer Nature.

Liu and colleagues introduced a comprehensive Raman spectroscopic system designed for non-invasive, transcutaneous glucose monitoring in both animal models and human subjects, and it exhibited a clear relationship between Raman spectral data and blood glucose concentrations, as shown in Figure 6. Specifically, the study utilized spectral intensity ratios at 125 cm^−1^ and 145 cm^−1^ as indicators [43]. It primarily concentrated on transcutaneous measurements. The research underscored the potential of Raman spectroscopy for non-invasive glucose monitoring, and it emphasized the use of specific Raman shifts for precise quantification. mµSORS demonstrated significant promise for clinical applications in the field of non-invasive blood glucose monitoring. However, additional refinement and miniaturization of the technology were required to employ it for AH-based NIGM applications.

#### 3.1.4. Advantages, Limitations, and Prospects for Clinical Translation

Raman spectroscopy provides numerous benefits for AH-based NIGM. We have summarized the reviewed studies in Table 1. This technique, which does not require labels, allows for a direct assessment of glucose concentration through the analysis of its distinctive molecular vibrations. The method is rapid and has the potential for real-time monitoring. Moreover, by targeting AH, it leverages a biofluid with relatively low interference compared to other tissues. However, Raman scattering is inherently a weak effect, which can pose challenges in achieving sufficient sensitivity for accurate glucose measurements at physiological concentrations. Developing robust calibration models that can account for individual variations in AH composition and potential interference from other components is also crucial.

Looking ahead, the prospects for the clinical translation of Raman spectroscopy for AH glucose detection are promising. It is crucial to obtain a strong Raman signal. This requires a highly sensitive Raman spectrometer. To facilitate clinical applications, the instrument must also be as compact as possible and with low power consumption. In addition, a suitable spectral resolution is essential to accurately distinguish the specific Raman peaks of glucose from other biomedical components. By achieving these characteristics of high sensitivity, small size, low power consumption, and suitable spectral resolution, Raman spectroscopy can become closer to widespread clinical application for AH glucose detection. The development of on-chip spectrometers in recent years has greatly promoted the practical application of AH-based Raman detection. We will review the progress of this technology in Section 4.

### 3.2. Using the Chiral Optical Rotation of Glucose for Aqueous Humor Glucose Detection

#### 3.2.1. Principles of Glucose Optical Rotation and Its Application in Concentration Determination

**Figure 7:**
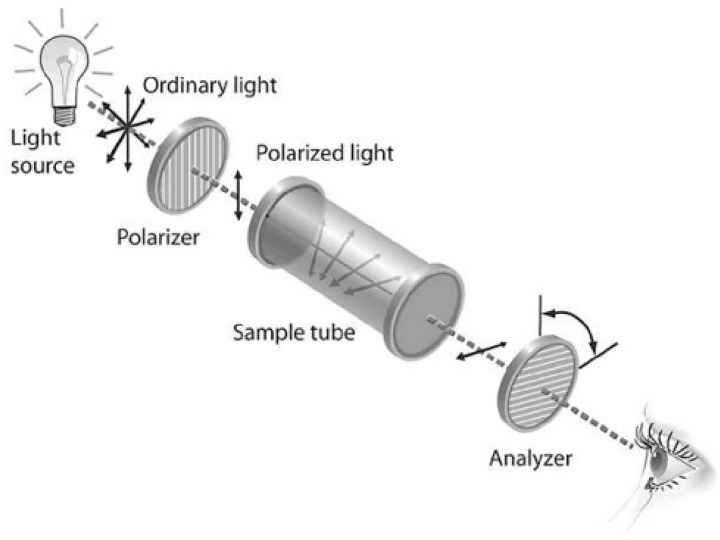
An example of plane-polarized light. Adapted from Ref. [56].

Glucose is a type of chiral molecule, characterized by its possession of a non-superimposable mirror image which arises due to the presence of one or more chiral centers within its structure. One of the defining features of chiral molecules is their capacity to rotate the plane of polarization in linearly polarized light, a phenomenon termed optical rotation or optical active. When linearly polarized light traverses a solution comprising a chiral compound such as glucose, the plane of its polarization undergoes a specific angular rotation, as shown in Figure 7. The magnitude of this rotation is directly proportional to the concentration of the chiral substance and the path length of the light through the solution, as well as the specific rotation of the substance, which is a characteristic property at a given temperature and wavelength of light. Polarimeters are instruments used to measure the angle of optical rotation. By measuring the optical rotation of AH, the glucose concentration can be determined, provided that the specific rotation of glucose is known and the contributions from other optically active substances are accounted for.

#### 3.2.2. Chiral Classification of Glucose in the Human Body (α- and β-Anomers)

In AH, glucose exists predominantly in a cyclic form, specifically as two diastereomeric anomers: α-D-glucose and β-D-glucose. These anomers differ in the configuration of the hydroxyl group at the anomeric carbon (C-1) [57]. The α-anomer has the hydroxyl group on the opposite side of the ring from the CH_2_OH group at C-5, while the β-anomer has it on the same side. These two anomers exhibit different specific rotations in water: α-D-glucose has a specific rotation of +112 degrees, and β-D-glucose has a specific rotation of +18.7 degrees [56]. When either pure anomer is dissolved in water, it undergoes mutarotation, a process where the α and β forms interconvert until an equilibrium is established. At equilibrium in water at 25 °C, the mixture contains approximately 36% α-D-glucose and 64% β-D-glucose, resulting in an observed specific rotation of +52.7 degrees. This equilibrium and the presence of both anomers must be considered for accurate glucose concentration measurements based on optical rotation. On the other hand, the optical rotation also shows wavelength dependence, which is shown in Table 2. This feature would help to compensate for motion-induced changes in corneal birefringence.

#### 3.2.3. Influence of Other Organic Substances in Aqueous Humor on Polarization Optical Rotation Properties

While glucose is the primary chiral molecule present in significant concentrations in AH, other organic substances, such as proteins and lactate, can also exhibit optical rotation and potentially contribute to the overall rotation of polarized light. Table 3 shows the optical active constituents in human AH [56].

To address the cumulative rotational effects of optically active compounds within AH, work has been performed to analyze the contributions of its primary chiral constituents: D-glucose, L-ascorbic acid, and albumin. Using their respective physiological concentrations and specific rotations, experiments have been conducted to calculate the total observed optical rotation [58,59,60].

The analysis confirms that the cumulative rotation is overwhelmingly dominated by D-glucose, which accounts for 94–97% of the total polarimetric signal, depending on the wavelength. The combined rotational effect from ascorbic acid and albumin is minimal, contributing a maximum potential error of just 6%. Although the influence of these other compounds is small, their presence is significant for precise measurements. Therefore, a multi-wavelength polarimetric approach is utilized. This technique effectively deconvolutes the signals from multiple chiral agents, which has been shown to reduce the glucose prediction error by a factor of two, thereby improving the measurement accuracy in complex biological samples [58].

#### 3.2.4. Influence of Corneal Birefringence on Quantitative Analysis of Detection

Corneal birefringence presents a notable obstacle in accurately quantifying the glucose concentration in AH through optical rotation methods [61,62]. Birefringence refers to the material property wherein the refractive index varies based on the polarization and direction of light propagation. The cornea displays birefringence due to its organized collagen structure, which can modify the polarization state of light as it traverses through. The alteration in polarization can obscure or disrupt the subtle rotation induced by glucose’s optical activity in AH, functioning as a notable noise factor in polarimetric glucose detection. Moreover, variations in corneal birefringence over time, caused by eye movements, further challenge the precision of glucose-induced rotation measurements. To tackle this problem, researchers have investigated various methods, such as dual-wavelength polarimetry [63]. This technique employs two distinct wavelengths of light to distinguish between the wavelength-dependent optical rotation of glucose and the relatively wavelength-insensitive birefringence of the cornea. Modeling corneal birefringence and optimizing the light beam’s path through the cornea are crucial strategies to minimize their impact on the accuracy of glucose detection utilizing optical rotation.

#### 3.2.5. Review of the Literature on Polarimetric Glucose Monitoring in Aqueous Humor

In 1982, initial studies investigated the possibility of utilizing AH glucose levels to ascertain the blood glucose concentration [64]. The authors created a model of a glucose sensor based on an optical bench, utilizing a pulsed laser along with a system comprising polarizers and detectors. They exhibited the capability to quantify glucose solutions and deliberated on the prospects of a non-invasive technique utilizing a scleral lens.

**Figure 8:**
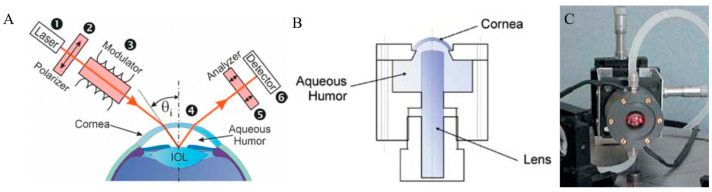
(**A**) Typical setup for polarimetric in vivo measurements. (**B**) Scheme of artificial eye. (**C**) Laboratory setup. Adapted with permission [65]. Copyright 2004, Springer Nature.

Rawer et al. [65] presented a high-resolution polarimetric measurement system in detail. It employed the optical rotatory dispersion phenomenon of glucose to determine its concentration in AH. The authors delved into the constraints associated with in vivo measurements, particularly highlighting the optical characteristics of the eye and eye movements. They suggested a novel approach utilizing a modified intraocular lens (IOL), depicted in Figure 8B,C, to enhance reflection and thereby facilitate more precise measurements.

**Figure 9:**
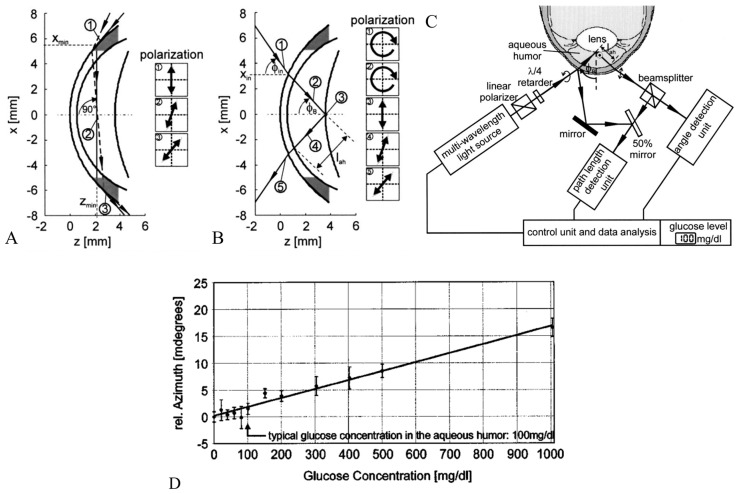
Optical access to the human eye. (**A**) Tangential path (solid beam path). The dashed beam-path indicates the first possible entrance condition. (**B**) New scheme applying Brewster reflection off the lens. (**C**) Diagram of the glucose sensor. (**D**) Measurement of the rotation of light polarization for different known glucose samples. Each measurement was repeated 50 times for statistical accuracy. The vertical error bars show the standard deviation of each measurement. The correlation coefficient of the linear regression amounts to R^2^ = 0.986. Adapted with permission [66]. Copyright 2004, SPIE.

Ansari et al. introduced a novel optical approach for polarimetric glucose sensing, leveraging the Brewster reflection of the eye lens, as depicted in Figure 9 [66]. The authors contended that this method offered greater accuracy compared to the tangential path approach. They provided a theoretical analysis alongside an experimental setup to confirm the efficacy of the proposed scheme and showcased its potential applications in non-contact glucose monitoring.

Corneal birefringence posed challenges for polarimetric techniques. As noted by Wan et al., their work (2015) tackled the issue of varying birefringence due to eye movements in polarimetric glucose sensing [63]. The authors created a dual-wavelength polarimetric system alongside an algorithm designed to counteract motion-induced birefringence. The in vitro findings indicated that the dual-wavelength method markedly decreased errors in comparison to single-wavelength systems.

Purvinis et al. described reports utilizing optical polarimetry to quantify glucose concentration in the AH of rabbits in vivo, as cited in [67]. The authors employed a specially developed laser-based polarimetry system and correlated its measurements with blood glucose concentrations. The findings indicated that polarimetric glucose monitoring was feasible in a living organism.

**Figure 10:**
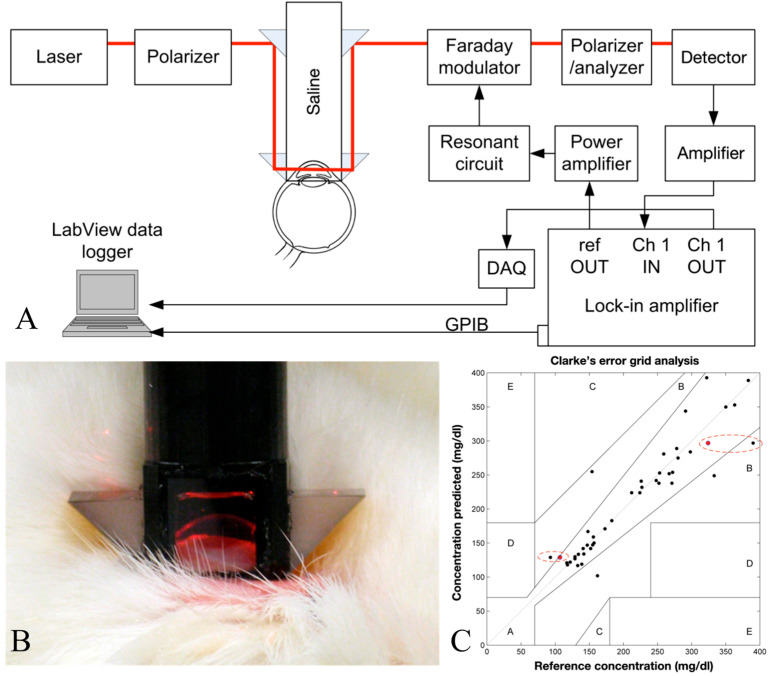
(**A**) The polarimetric experimental setup employed for the sensing glucose concentration in the eye. DAQ, data acquisition; GPIB, general purpose interface bus. (**B**) The coupling of the glucose-sensing optical signal through the aqueous humor of a NZW rabbit. (**C**) Clarke error grid showing 41 points, with 93% in region A and the remainder in region B. The reference concentrations are from a handheld glucometer (One Touch Ultra, Lifescan Inc., Milpitas, CA, USA), except for circled points. Circled points additionally have YSI-measured concentrations as reference (red points) and show the predicted values are closer to the YSI measurements than the handheld meter. Adapted with permission [67]. Copyright 2011, Sage.

Hwang et al. demonstrated the progression of an NIGM designed to quantify AH glucose concentrations in rabbits [68]. Their demonstration employed a hybrid optical system, integrating near-infrared absorption measurements with polarized rotatory distribution assessments, as shown in Figure 10. The authors conducted both in vitro and in vivo experiments to demonstrate the device’s precision and its correlation with serum glucose levels.

**Figure 11:**
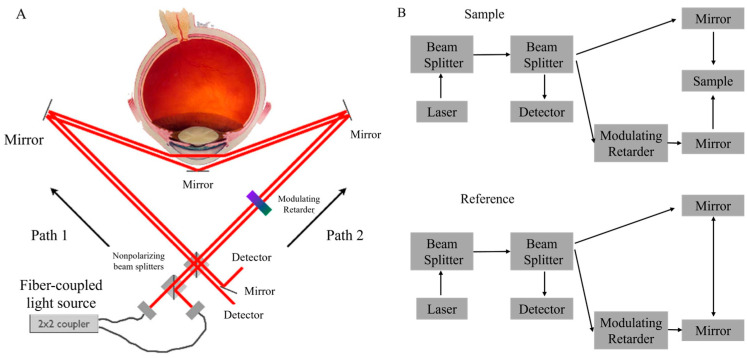
(**A**) Schematic of a Sagnac interferometer, where light from counterpropagating paths is combined. For a stationary interferometer with no sample, the optical paths are identical. Optical rotation, however, introduces an optical path difference between the counterpropagating beams. The modulating retarder, which continuously changes the optical rotation from 0 to 360 deg, produces a cosinusoidal interference signal at the detector. An additional optical rotation from the eye results in a phase difference between the reference and sample signals. (**B**) The optical path of the sample and reference beam. Adapted from [69].

A Sagnac interferometer was utilized in reference [69]. The Sagnac interferometry method is advantageous for glucose sensing due to its sensitivity to optical rotation while exhibiting insensitivity to net linear birefringence and the alignment of the incident polarization state, which posed significant challenges for conventional methods. The setup’s schematic was depicted in Figure 11A. The paper emphasized the significance of laser wavelength, noting that the specific rotation of glucose was contingent upon both wavelength and temperature [52]. The research cited indicated that variation with temperature was insignificant at wavelengths exceeding 700 nm. The study’s simulation employed wavelengths of 700 nm, 750 nm, and 800 nm. This range was chosen due to the negligible influence of confounders on glucose in AH beyond 700 nm. The paper described an experiment that employed a fiber-coupled superluminescent diode, with a central wavelength of 830 nm, as the light source. The specific rotation of glucose diminished as the wavelength escalated, indicating that a wavelength near 700 nm was optimal for glucose measurements. Nonetheless, attenuation arising from Fresnel reflections at the corneal surface persisted as a confounding factor that might have necessitated refractive-index matching for mitigation.

#### 3.2.6. Advantages, Limitations, and Prospects for Clinical Translation

Polarimetric methods show promise for non-invasive glucose monitoring due to their potential for high resolution, sensitivity, and feasibility for miniaturization. We have summarized the reviewed studies in Table 4. Research has demonstrated a good correlation between AH and blood glucose levels, and the technology can potentially be integrated into wearable devices like contact lenses. However, challenges remain, including the interference from other optically active substances and errors caused by corneal birefringence and eye movements.

Recent advancements, such as multi-wavelength polarimetry and specialized IOLs, are being explored to address these limitations and improve accuracy. If these challenges can be overcome, polarimetry-based glucose monitoring could offer a valuable tool for more convenient and effective diabetes management.

### 3.3. Using Mid-Infrared and Near-Infrared Absorption Spectra for Aqueous Glucose Detection

#### 3.3.1. Principles of Using Mid-Infrared and Near-Infrared Absorption Spectra for Aqueous Glucose Detection

Infrared (IR) spectroscopy operates on the principle that molecules absorb light at distinct frequencies, which correspond to their vibrational modes. Glucose molecules display unique absorption patterns in both the mid-infrared (MIR) and near-infrared (NIR) regions of the electromagnetic spectrum. Specifically, MIR spectroscopy is utilized to analyze these absorption characteristics. Typically, this covers the range of 2.5–25 µm (4000–400 cm^−1^). This encompasses the “fingerprint region” spanning from 8.33 to 12.5 µm (1200 to 800 cm^−1^), where glucose exhibits unique and pronounced absorption peaks, providing a high degree of specificity for glucose detection [70,71,72,73,74]. Nonetheless, the penetration depth of MIR light in biological tissues is limited. Specifically, in aqueous environments, there is significant water absorption, making NIR spectroscopy particularly relevant. Within the spectrum of 700 to 2500 nm, it provides superior penetration depth in tissues, and water exhibits greater transparency in this spectral range. Additionally, glucose has absorption bands within the NIR region, despite being generally weaker and broader compared to those in the MIR. Glucose-specific signals are often extracted using multivariate analysis, accounting for background noise and interfering substances. Non-invasive glucose monitoring via IR absorption spectroscopy involves directing IR light through or reflecting it off the target tissue or fluid. By measuring the intensity of transmitted or reflected light at specific wavelengths, glucose concentration can be determined based on the Beer–Lambert Law [75].

#### 3.3.2. Review of the Recent Literature on MIR/NIR Absorption for Aqueous Glucose Detection

Wolfgang et al. investigated the feasibility of utilizing near-infrared (NIR) spectroscopy for non-invasive glucose measurement in the human eye [76]. The research introduced a well-organized methodology for examining the application of NIR spectroscopy in non-invasive glucose measurement within the eye. The in vitro experiments established a basis for comprehending the spectral characteristics of glucose in AH and facilitated the development of a predictive model. The employment of chemometric analysis and Clarke error grid analysis strengthened the reliability and practical significance of the research findings. The initial in vivo findings suggested limitations in accuracy, potentially attributed to patient movement and the low reflectivity of the eye lens. The authors acknowledged the limitations and proposed potential solutions, including the use of lasers and enhanced geometrical control, to improve the method’s reliability. The research indicated that, although the method demonstrated promise, additional enhancements were necessary to attain the necessary precision for dependable clinical use.

Hwang et al. presented the evolution of an NIGM designed to quantify AH glucose levels in rabbits [68]. They integrated measurements of near-infrared absorption and polarized rotatory distribution, demonstrating notable advantages.

#### 3.3.3. Advantages, Limitations, and Prospects for Clinical Translation

Future research may focus on developing more sensitive spectroscopic techniques, employing specific wavelengths with minimal water absorption, and using advanced data processing methods to enhance the signal-to-noise ratio and improve accuracy. Combining absorption spectroscopy with other optical techniques, such as polarimetry or PAS, might also offer synergistic benefits for AH-based NIGM.

Like Raman spectroscopy, a portable, sensitive, and fast spectrometer is needed in this sensing scenario and would enable a convenient AH-based NIGM. We will review the progress of this technology in Section 4.

### 3.4. Other Potential Optical Method for Aqueous Glucose Detection

PAS and OCT represent two distinct optical methodologies with potential applications in glucose sensing. PAS operates on the principle of the photoacoustic effect: pulsed laser light is absorbed by the sample, leading to localized heating and subsequent generation of ultrasonic waves that can be detected. The amplitude of these acoustic waves is proportional to the absorbed optical energy, providing information about the sample’s optical absorption spectrum. The current applications of PAS span various fields, including label-free molecular imaging of biological tissues and the detection of specific analytes in vitro [77,78,79,80]. OCT, on the other hand, is an interferometric technique that utilizes low-coherence light to generate high-resolution, cross-sectional images of biological tissues based on their refractive index variations. Its primary applications lie in ophthalmology for retinal imaging and anterior segment analysis, as well as in guiding surgical procedures [81,82,83].

Despite their advancements, the direct in vivo measurement of AH glucose concentration using PAS and conventional OCT faces significant challenges. PAS sensitivity can be limited by the low glucose concentration in AH and potential interference from other absorbing molecules. While OCT excels in structural imaging, its direct sensitivity to glucose concentration is inherently low, requiring indirect approaches or the use of exogenous contrast agents that might be invasive or unstable in the ocular environment.

Future efforts to adapt PAS for AH glucose monitoring could explore the use of highly sensitive acoustic transducers and excitation wavelengths optimized for glucose absorption while minimizing interference. For OCT, research might focus on developing functional OCT techniques that are sensitive to glucose-induced changes in the refractive index or on integrating specific glucose-sensitive nanoparticles or hydrogels that can be detected with high spatial resolution. Exploring multi-modal approaches combining the strengths of both PAS and OCT could also offer promising avenues for AH-based NIGM.

## 4. Recent Development of Compact Spectrometer

In previous sections, we have comprehensively reviewed various optical methodologies for NIGM by using AH as the biomedical media. Among these promising techniques, Raman spectroscopy and NIR spectroscopy have emerged as particularly significant due to their inherent potential for AH-based NIGM. However, the successful translation of both Raman and NIR spectroscopy into practical applications is critically reliant on the availability of suitable, high-performance instrumentation. A clear and urgent requirement exists for NIR spectrometers that offer both high accuracy in capturing spectral features and a compact, miniaturized design suitable for clinical and wearable applications. Consequently, this section examines recent progress in compact, power-efficient spectrometers applicable to AH-based NIGM. We will first review the conventional approaches, then explore the recent innovations in on-chip spectrometer technology, crucial for realizing compact, portable, and wearable sensing systems for AH-based NIGM.

### 4.1. Conventional NIR Spectrometer Technologies

Before exploring on-chip solutions, we will first review the working principle of the conventional NIR spectrometers. They often offer high performance, while typically facing challenges in terms of size, cost, and suitability for integration into portable devices.

#### 4.1.1. Dispersive Spectrometers

**Figure 12:**
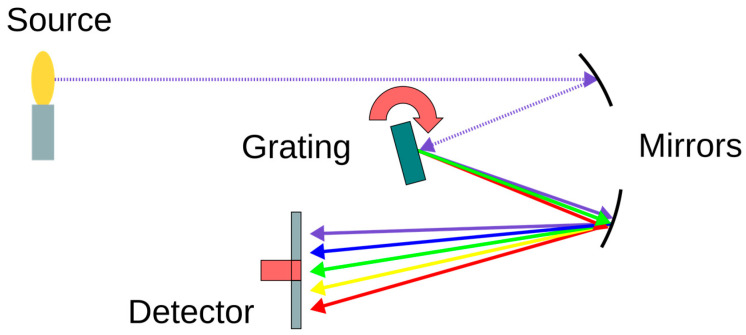
Schematic of a traditional dispersive spectrometer. Adapted from [84].

Traditional dispersive spectrometers are a foundational technology in spectroscopic analysis. Their operational principle involves the spatial separation of polychromatic light into its individual wavelengths. This separation is typically accomplished using a dispersive element, such as a diffraction grating or a prism, as shown in Figure 12.

The process begins with incident light, which is often passed through an input slit to define the light beam and improve spectral resolution. This light is then collimated, subsequently dispersed by the grating or prism, and finally focused on a detector array. For NIR region analysis, an Indium Gallium Arsenide (InGaAs) detector array is commonly employed. Each element within this array measures the intensity of a specific, narrow band of wavelengths, enabling the reconstruction of the complete spectrum.

Dispersive spectrometers represent a mature technology capable of delivering high spectral resolution (sub-nanometer) across a broad wavelength range. However, a significant drawback is their physical size, which is inherently linked to the focal lengths required for collimating and focusing optics, as well as the dimensions of the dispersive elements and the detector array. Furthermore, achieving a high spectral resolution necessitates the use of narrow input slits. While effective for resolution, this approach curtails light throughput, potentially leading to longer integration times or the need for more sensitive (and consequently, more expensive) detectors. These collective limitations render traditional dispersive spectrometers less suitable for applications such as AH-based NIGM.

#### 4.1.2. Filter-Based Spectrometers

**Figure 13:**
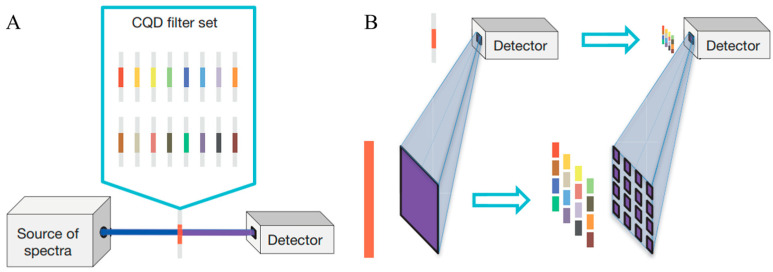
(**A**) A basic quantum dot spectrometer is composed of a set of CQD absorptive filters and a light detector. (**B**) Instead of measuring the light intensities using one detector and one filter at a time, the CQD spectrometer measures the set of intensities in parallel by using an array detector, with each detecting element dedicated to one CQD filter, all of which are integrated into a CQD filter array. Adapted with permission [85]. Copyright 2015, Springer Nature.

Filter-based spectrometers employ a set of optical filters, each designed to transmit a specific wavelength band, to select and measure light intensity at discrete wavelengths. These can range from simple colored glass filters to more sophisticated interference filters or electronically tunable filters like Liquid Crystal Tunable Filters (LCTFs) or Acousto-Optic Tunable Filters (AOTFs), and some of the recent designs even involve using colloidal quantum dots (CQDs) [85], as shown in Figure 13. The light passes sequentially or in parallel through these filters, and the intensity of the transmitted light for each filter is measured by a single photo detector or an array. They have advantages such as simplicity, robustness and low cost compared to other technologies. However, the most significant limitation is their inherently low spectral resolution, which is determined by the bandwidth of the individual filters. They are generally not suitable for AH-based NIGM application cases.

#### 4.1.3. Fourier Transform Spectrometers

**Figure 14:**
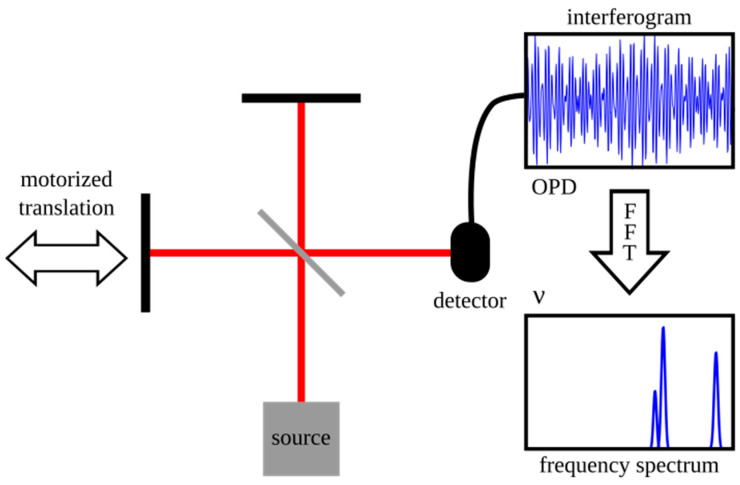
Schematic of a Fourier transform spectrometer. Adapted from Ref. [87].

Fourier transform spectrometry (FTS) is a powerful interferometric technique widely employed for acquiring spectral information [86]. At its core, a typical FTS system utilizes a Michelson interferometer, as shown in Figure 14. In this setup, incoming light is directed onto a beamsplitter, which divides it into two separate beams. One beam traverses a fixed path length, while the path length of the other beam is systematically varied by a precisely controlled moving mirror. These two beams are subsequently recombined at the beamsplitter, creating an interference pattern. This pattern, known as an interferogram, is recorded by a single-element detector as a function of the optical path difference (OPD) between the two beams. The crucial final step involves performing a Fourier transform on this measured interferogram to yield the spectrum of the incident light.

FTS instrumentation presents three primary and distinct advantages over traditional dispersive spectrometers [86]:

Felgett’s Advantage (Multiplex Advantage): In an FTS instrument, all wavelengths of the input light reach the detector simultaneously throughout the entire measurement period, rather than being scanned and observed sequentially as in dispersive systems. This parallel detection significantly enhances the signal-to-noise ratio (SNR), particularly in scenarios where detector noise is a limiting factor. Consequently, measurements can often be performed more rapidly or with greater sensitivity.

Jacquinot’s Advantage (Throughput Advantage): Unlike dispersive spectrometers that necessitate narrow slits to achieve high spectral resolution (which inherently limits the amount of light passing through), FTS systems do not have this constraint. They can accept a larger input aperture for a given resolution, resulting in a significantly higher optical throughput. This increased light-gathering capability is especially beneficial when analyzing weakly emitting or absorbing samples, where maximizing the signal is critical.

Connes’ Advantage (Wavelength Accuracy): The wavelength scale in FTS is intrinsically and highly accurately calibrated. This is achieved by co-propagating a reference laser, typically a helium–neon (HeNe) laser with a precisely known wavelength, with the signal beam. The interferogram of this reference laser provides a highly accurate and precise measure of the OPD, ensuring exceptional wavelength accuracy and precision in the resulting spectrum. The reference laser also plays a role in precisely timing the movement of the mirror and the sampling of the interferogram.

Despite these significant benefits, traditional FTS systems face limitations, primarily stemming from the requirement for precise, long-range, and stable movement of the mirror. This mechanical necessity can render the instruments sensitive to environmental vibrations, potentially impacting measurement accuracy and reliability. Furthermore, the sophisticated mirror-moving mechanism can contribute to a larger instrument footprint and increased manufacturing costs.

### 4.2. On-Chip NIR Spectrometer Technologies

The limitations of conventional spectrometers in terms of size, cost, and robustness have driven the development of on-chip spectroscopic solutions, leveraging microfabrication techniques from the semiconductor and photonics industries. These integrated devices offer the potential for mass production, significantly reduced footprints, and enhanced stability.

#### 4.2.1. On-Chip Dispersive Spectrometers

Miniaturized dispersive spectrometers strive to replicate the functionality of their larger, bulk-optic counterparts on a photonic integrated circuit (PIC). Common approaches utilize planar waveguides to guide light and incorporate specially designed structures for wavelength dispersion. Arrayed waveguide gratings (AWGs) [88] and planar concave gratings (PCGs) [89], initially well-established in optical telecommunications for wavelength division multiplexing, are readily adaptable for spectroscopic applications. These devices can be fabricated using standard lithographic techniques on various material platforms, such as silicon-on-insulator (SOI), silica, or silicon nitride (SiN), enabling the creation of compact and robust spectrometers.

However, a significant limitation of these approaches is their relatively narrow operational bandwidth, which is constrained by the free spectral range (FSR) [88]. Achieving high resolution necessitates a large optical path length difference within the dispersive element, a characteristic that often results in a smaller FSR and thus a more limited bandwidth. While cascading multiple AWGs or PCGs can extend the overall operational bandwidth, this solution introduces additional complexity and can potentially increase optical losses. Furthermore, the performance of these miniaturized spectrometers can be sensitive to fabrication tolerances and temperature variations.

#### 4.2.2. On-Chip Fourier Transform Spectrometers

On-chip FTS architecture presents a promising technological pathway, potentially retaining the significant etendue and multiplex advantages inherent to FTS while overcoming the physical scale limitations of conventional benchtop systems. In these integrated designs, the function of the macroscopic moving mirror is replicated by creating precisely controlled optical path length differences within waveguide interferometer structures. These path length differences can be dynamically modulated, often through thermo-optic or electro-optic means, obviating the need for mechanical actuation. Recent breakthroughs in on-chip FTS demonstrate its substantial potential for biomedical sensing, with notable applicability in areas such as AH-based NIGM.

##### Spatial Heterodyne Spectrometers

**Figure 15:**
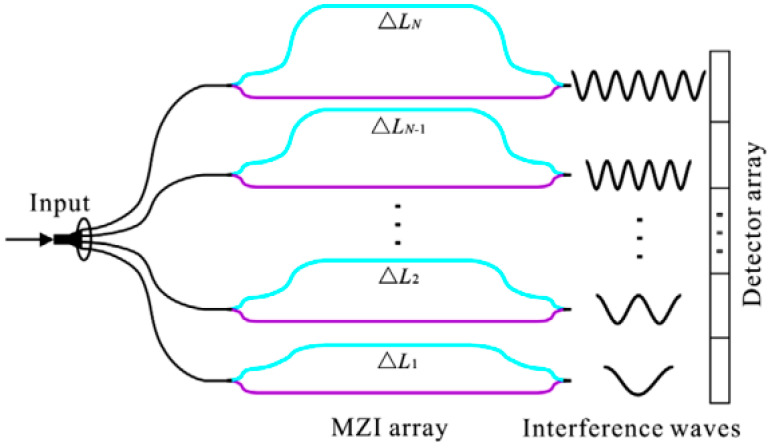
Schematic of planar waveguide on-chip SHS formed by an array of imbalanced MZIs. Adapted with permission [91]. Copyright 2021, John Wiley and Sons.

Typical spatial heterodyne spectrometers can be realized using an array of unbalanced MZIs. In this setup, each MZI introduces a phase delay, causing light to interfere with a delayed version of itself. Crucially, the OPD across these MZIs increases in fixed steps from the first to the Nth channel, as shown in Figure 15. The resulting intensity variations at the linearly arranged output ports of the MZI array create a spatially dependent, stationary interference pattern. This interferogram is then recorded by a photodiode array. In this method, enhancing spectral resolution across a given bandwidth requires an increased channel count. This, in turn, leads to a rapid expansion of the device’s physical dimensions as the spectral resolution is scaled upwards [90]. SHS offers a path to very high resolution, albeit typically with an increased device footprint and a significant limitation in bandwidth. The reported maximum bandwidth for SHS systems, around 60 nm, falls short of covering the full NIR range. Addressing the inherent difficulties in fabricating consistent MZI arrays at the wafer level, calibration algorithms are employed to mitigate adverse effects like insertion and propagation losses, as well as phase inaccuracies.

##### Stationary Wave Integrated Fourier Transform Spectrometers

The on-chip Stationary Wave Integrated Fourier Transform Spectrometer (SWIFTS) represents a notable evolution within the FTS family, facilitating the development of compact, efficient, and static (no moving parts) spectrometers. Its operational principle relies on detectors interacting with the evanescent field of light guided via dielectric integrated optics [92].

A significant merit of SWIFTS technology is its minimal physical footprint, with chip lengths typically confined to a few millimeters. Nevertheless, these devices provide a wide array of spectral optical detection capabilities, including high spectral resolutions (on the order of nanometers or less) and operational bandwidths spanning 100 to 200 nanometers. Such characteristics render SWIFTS particularly advantageous for applications like AH-based NIGM.

**Figure 16:**
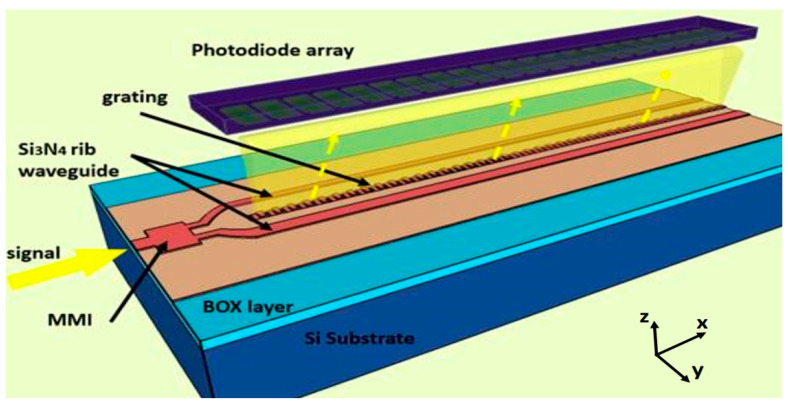
Schematic of grating-assisted SWIFTS. Adapted with permission [93]. Copyright 2017, Optica Publication Group.

The progression of SWIFTS technology has been consistent. Initial demonstrations in 2007 reported a 4 nm resolution across a 96 nm bandwidth [92]. Subsequently, Xiaomin and colleagues showcased a fully CMOS-compatible SWIFTS design, as shown in Figure 16, with provision for on-chip photodetector integration, which demonstrated a 6 nm resolution over a 100 nm bandwidth within a 0.1 mm^2^ footprint [93]. Further enhancements yielded resolutions as fine as 0.025 nm over a 256 nm bandwidth, signifying the technology’s capacity for highly demanding spectral analysis tasks [94].

##### Temporally Modulated Static Fourier Transform Spectrometers

The key component in these temporally modulated static FTS systems is an integrated interferometer, commonly realized using a Mach–Zehnder Interferometer (MZI) or, in some designs, a micro-ring filter (MRF) [95]. The critical feature of these devices is the deliberate and precisely controlled variation, or temporal modulation, of the OPD between different optical paths. This modulation is frequently accomplished by integrating a thermo-optic (TO) heater with one arm of the MZI or MRF. Altering the temperature of this specific arm modifies its refractive index, thereby changing its optical path length and inducing a time-varying OPD.

As the OPD is systematically scanned, different wavelengths within the input light undergo constructive and destructive interference at the device’s output. This fluctuating interference pattern, known as an interferogram, is captured by a single photodetector or several photodetectors as a time-varying signal. Subsequently, a mathematical Fourier transform is applied to the temporal interferogram. This computational step reconstructs the original optical spectrum of the input light, revealing the intensity of its constituent wavelengths. This approach facilitates the creation of robust and compact spectrometer designs.

Early pioneering demonstrations of such on-chip FTS devices were predominantly carried out on silicon-on-insulator (SOI) platforms. Silicon’s high thermo-optic efficiency is a significant advantage, allowing for effective OPD tuning with relatively low power consumption. For instance, research by Ang and colleagues, in 2023, showcased a temporally modulated FTS employing an MZI architecture on an SOI platform [96]. Their device reportedly achieved a spectral resolution of approximately 0.1 nanometers over a 100-nanometer bandwidth. However, a notable limitation of silicon-based devices is the material’s absorption of light at wavelengths below 1100 nm, preventing full coverage of the near-infrared (NIR) spectrum, which is crucial for our AH-based NIGM applications.

**Figure 17:**
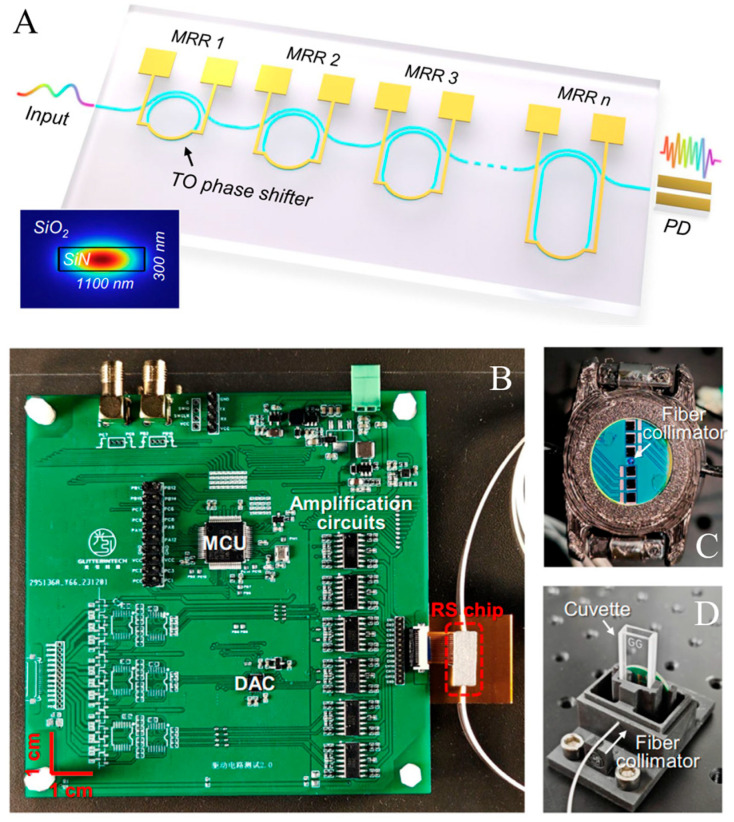
(**A**) Conceptual schematic of the proposed ultra-broadband spectrometer featuring multiple stages of micro-ring resonators on a single bus. The inset shows the mode field distribution on the bus waveguide. (**B**) Photograph of the miniaturized near-infrared spectrometric sensor. The insets show the optical sampling interfaces for measuring reflection and transmission spectra, respectively. MCU, microcontroller unit. DAC, digital-to-analog converter. (**C**) Fiber collimator of the spectrometer. (**D**) The stage for the cuvette and fiber collimator. Adapted with permission [97]. Copyright 2024, Springer Nature.

To address this limitation, designs utilizing silicon nitride (SiN) have emerged as a promising alternative, as shown in Figure 17. Although SiN has a much lower thermo-optic efficiency, it offers a significantly larger transparent window, extending across a broader range of wavelengths. Illustrating this advantage, Chunhui et al. demonstrated similar spectrometer designs incorporating cascaded MRFs on a SiN platform [97]. Leveraging SiN’s broad transparency, their spectrometer achieved an impressive 520-nanometer bandwidth with a fine resolution of below 8 picometers.

### 4.3. Conclusions and Perspective

The evolution of these compact spectrometers directly addresses key challenges in deploying Raman and NIR spectroscopy for AH NIGM, such as the need for high sensitivity, selectivity, and user-friendly operation. As shown in Table 5, these spectrometer technologies continue to mature, becoming more cost-effective and powerful, their integration into clinically viable therapies appears increasingly feasible, promising a significant improvement in diabetes management.

## 5. Conclusions and Future Directions: Towards Advanced Non-Invasive Glucose Monitoring Systems

Our comprehensive review of optical techniques solidifies the primary finding that AH stands out as an exceptionally promising medium for non-invasive glucose monitoring. Its optical accessibility and direct physiological link to blood glucose levels provide a unique window for reliable, continuous measurement. The various methods discussed, including Raman spectroscopy, polarimetry, and NIR spectroscopy, all leverage this unique anatomical feature.

The central conclusion of this review is the significant, yet largely untapped, potential for a multi-modal sensing approach. Since these distinct optical methods can all share the same light path through AH, a powerful opportunity exists to merge them into a single, integrated sensor. In such a device, Raman spectroscopy, polarimetry, and NIR absorption would not merely operate in parallel but could serve as mutual calibrants. This cross-calibration capability is a critical advantage, as the chemical specificity of Raman, the chirality sensitivity of optical rotation, and the penetration depth of NIR can be synergistically combined to overcome the limitations of any single method, thereby significantly enhancing measurement accuracy and robustness against interference.

The clinical translation of such an integrated sensor is no longer a distant prospect, thanks to a pivotal technological enabler: the rapid advancement in integrated, miniaturized spectrometers. The development of compact, precise, and low-power spectral analyzers is crucial for making a multi-modal AH sensor a practical reality. These next-generation spectrometers are increasingly capable of performing the high-sensitivity measurements required for both Raman and NIR spectroscopy within a form factor suitable for a wearable or portable device. By harnessing these technological strides, future research can focus on developing a unified system that redefines the standards of non-invasive glucose detection. This integrated approach, grounded in the unique properties of AH, holds the definitive potential to deliver a clinically viable solution that can finally free patients from the pain and inconvenience of invasive glucose monitoring.

## Figures and Tables

**Table 1:** Summary of reviewed research on Raman spectroscopy for glucose detection.

Reference	Sensitivity	Pumping Wavelength Detection Method	Consistency with Venous Sample
Wicksted et al. 1994 [52]	N/A	514.5 nm Single-grating monochromator and liquid-nitrogen-cooled CCD	Not reported
Pelletier et al. 2005 [53]	2.1–43 mmol/L	785 nm KOSI confocal Raman microscope	In vitro, RMSEP = 20.4 mg/dL, r^2^ = 0.991 (HAH vs. AAH model)
Sideroudi et al. 2006 [54]	11 mmol/L	514.5 nm CCD-based Raman spectroscopic system	N/A (in vitro)
Yang et al. 2018 [55]	0.1 to 30 mmol/L	785 nm Not specified	In vivo rabbit eyes, Accuracy within ± 0.5 mM of commercial glucose sensor
Liu et al. 2025 [43]	in vivo human, MARD of 14.6%, 99.4% of predictions in CEG A + B zones	785 nm Not specified	In vivo human, MARD of 14.6%, 99.4% of predictions in CEG A + B zones

**Table 2:** Specific rotation of glucose at various wavelengths of light. Adapted from Ref. [56].

Wavelength (nm)	447	479	508	535	589	659
Specific rotation °/(dm g/mL)	96.6	83.9	73.6	65.4	52.8	41.9

**Table 3:** Optically active constituents of human aqueous humor. Adapted from Ref. [56].

Constituent	Aqueous (mmol/L)	Plasma (mmol/L)	Specific Rotation
Ascorbic acid	1.1	0.04–0.06	24.00
Lactic acid	4.5	0.5–1.9	−2.226
Citric acid	0.12	0.12	
Glucose	2.7–3.9	5.6–6.4	52.7
Alanine	0.307	0.326	2.7
Arginine	0.105	0.071	12.5
Cysteine	0.015	0.134	9.8
Glutamine	0.010	0.058	6.5
5Histidine	0.069	0.078	−39.01
Isoleucine	0.066	0.053	11.29
Leucine	0.139	0.104	−10.8
Lysine	0.167	0.254	14.6
Methionine	0.059	0.024	−8.11
Phenylalanine	0.095	0.048	−35.14
Proline	0.047	0.231	−85.00
Serine	0.825	0.660	−6.83
Threonine	0.132	0.114	28.4
Tyrosine	0.094	0.053	
Valine	0.286	0.216	6.42
Total protein (mg/dL)	12.4–23.4	7000	
Albumin (mg/dL)	5.5–6.5	3400	−59–109

**Table 4:** Summary of reviewed research on polarimetric glucose monitoring in AH.

Reference	Technical Solution	Glucose Detection (mmol/L)	Laser Wavelength (nm)
Rabinovitch et al. (1982) [64]	Optical bench model; Scleral lens concept	1.1–5.5	904
Rawer et al. (2004) [65]	High-resolution polarimetry; Modified IOL	Not explicitly specified	400, 780
Ansari et al. (2004) [66]	Brewster reflection; Multi-wavelength approach	0–27.8 in solution	543,633
Wan et al. (2005) [63]	Dual-wavelength polarimetry; Algorithm for motion-induced birefringence	0–33.3	523, 635
Purvinis et al. (2011) [67]	Faraday-based optical polarimetry	5.2–28.9	632.8
Hwang et al. (2021) [68]	Hybrid system: NIR absorption and polarimetry; Alignment system	2.8–33.3	1650
Winkler et al. (2011) [69]	Sagnac interferometry	Not specified	Superluminescent diode centered at 830

**Table 5:** Summary of recent development of compact NIR spectrometer.

Reference	Spectral Resolution (nm)	Bandwidth (nm)	Size (mm^2^)	Center Wavelength (nm)
Rabinovitch et al. (2007) [92]	4	96	1	1550
Xiaomin et al. (2017) [93]	6	100	0.1	895
Mohammad et al. (2018) [94]	0.025	256	120	633
Ang et al. (2023) [96]	0.1	100	~4	1550
Chunhui et al. (2024) [97]	0.008	520	~5	1440

## Data Availability

Not applicable due to it is a review.
